# *Brugia malayi* Antigen (BmA) Inhibits HIV-1 *Trans*-Infection but Neither BmA nor ES-62 Alter HIV-1 Infectivity of DC Induced CD4^+^ Th-Cells

**DOI:** 10.1371/journal.pone.0146527

**Published:** 2016-01-25

**Authors:** Emily E. I. M. Mouser, Georgios Pollakis, Maria Yazdanbakhsh, William Harnett, Esther C. de Jong, William A. Paxton

**Affiliations:** 1 Laboratory of Experimental Virology, Department of Medical Microbiology, Centre for Infection and Immunity Amsterdam (CINIMA), Academic Medical Center, University of Amsterdam, Amsterdam, The Netherlands; 2 Department of Clinical Infection, Microbiology and Immunology (CIMI), Institute of Infection and Global Health, University of Liverpool, Liverpool, United Kingdom; 3 Department of Parasitology, Leiden University Medical Centre, Leiden, The Netherlands; 4 Strathclyde Institute of Pharmacy and Biomedical Sciences, University of Strathclyde, Glasgow, United Kingdom; 5 Department of Cell Biology and Histology, Academic Medical Center, University of Amsterdam, Amsterdam, The Netherlands; Vita-Salute San Raffaele University School of Medicine, ITALY

## Abstract

One of the hallmarks of HIV-1 disease is the association of heightened CD4^+^ T-cell activation with HIV-1 replication. Parasitic helminths including filarial nematodes have evolved numerous and complex mechanisms to skew, dampen and evade human immune responses suggesting that HIV-1 infection may be modulated in co-infected individuals. Here we studied the effects of two filarial nematode products, adult worm antigen from *Brugia malayi* (BmA) and excretory-secretory product 62 (ES-62) from *Acanthocheilonema viteae* on HIV-1 infection *in vitro*. Neither BmA nor ES-62 influenced HIV-1 replication in CD4^+^ enriched T-cells, with either a CCR5- or CXCR4-using virus. BmA, but not ES-62, had the capacity to bind the C-type lectin dendritic cell-specific intercellular adhesion molecule-3-grabbing non-integrin (DC-SIGN) thereby inhibiting HIV-1 *trans*-infection of CD4^+^ enriched T-cells. As for their effect on DCs, neither BmA nor ES-62 could enhance or inhibit DC maturation as determined by CD83, CD86 and HLA-DR expression, or the production of IL-6, IL-10, IL-12 and TNF-α. As expected, due to the unaltered DC phenotype, no differences were found in CD4^+^ T helper (Th) cell phenotypes induced by DCs treated with either BmA or ES-62. Moreover, the HIV-1 susceptibility of the Th-cell populations induced by BmA or ES-62 exposed DCs was unaffected for both CCR5- and CXCR4-using HIV-1 viruses. In conclusion, although BmA has the potential capacity to interfere with HIV-1 transmission or initial viral dissemination through preventing the virus from interacting with DCs, no differences in the Th-cell polarizing capacity of DCs exposed to BmA or ES-62 were observed. Neither antigenic source demonstrated beneficial or detrimental effects on the HIV-1 susceptibility of CD4^+^ Th-cells induced by exposed DCs.

## Introduction

Human immunodeficiency virus type 1 (HIV-1) is endemic in many regions of the world where individuals are infected with an array of pathogens [1]. Parasitic helminths infect large numbers of the population and are known to evade, skew and dampen immune responses thereby potentially having consequences for HIV-1 disease, as continuous immune system activation is one of the driving factors in HIV-1 infection and disease course [2]. Worldwide over 120 million people are infected with filarial nematodes, which can survive for decades within locations such as our lymphatic system and can cause high levels of morbidity [3,4]. How parasites and parasite antigens modulate HIV-1 infection, and vice versa, is relatively unknown but will have significance in regions where co-infection rates are high.

HIV-1 cell entry is facilitated by the gp120 envelope protein, which binds to its primary receptor CD4 and subsequently to a co-receptor, usually CCR5 or CXCR4 [5,6]. Direct infection of cells via these receptors is termed *cis*-infection. Additionally, gp120 can bind several C-type lectin receptors (CLR) on cells derived from the myeloid cell lineage. These cells can subsequently pass the virion to activated CD4^+^ T-lymphocytes, a process termed *trans*-infection [7]. Dendritic cell-specific intercellular adhesion molecule-3-grabbing non-integrin (DC-SIGN) is a CLR expressed on dendritic cells (DCs) known to support HIV-1 *trans*-infection and has been implicated in HIV-1 transmission and dissemination [7,8]. In addition to gp120, DC-SIGN can bind many glycosylated structures including a large array of pathogen-derived glycoproteins as well as numerous host proteins found in bodily secretions. Binding of these glycoproteins to DC-SIGN can actually block its ability to capture and transfer HIV-1 [9–12].

The main target cells for HIV-1 are CD4^+^ effector memory T-cells, also known as the T helper (Th) cells. Th-cells are subdivided based on their cytokine expression profile into three main types of effector Th-cells namely Th1, Th2 and Th17 cells which are induced by intracellular pathogens (e.g. viruses), parasites and bacterial or fungal infections, respectively [13]. The balance between HIV-1 co-receptor expression and production of their natural ligands, MIP-1α, MIP-1β and RANTES for CCR5, and SDF-1 for CXCR4 has been shown to be important in determining T-cell susceptibility to HIV-1 infection [14–16]. More recent studies have correlated pathogen-specific CD4^+^ T-cells to HIV-1 susceptibility [17–19]. Geldmacher et al correlated variation in HIV-1 susceptibility with a difference in the cytokine/chemokine profile of CD4^+^ T-cells. They demonstrated that *Mycobacterium tuberculosis* specific-CD4^+^ T-cells, lost rapidly after HIV-1 infection, express high levels of IL-2 and low levels of MIP-1β contrary to the CMV specific CD4^+^ T-cells, which possess the reverse profile and typically survive until end stage AIDS [17]. Besides cytokines, heightened levels of T-cell activation have been associated with the up regulation of numerous HIV-1 restriction factors thereby potentially modulating cellular infection profiles [20].

Parasitic helminths are known for their capacity to induce T-cell responses with a Th2 phenotype. In previous studies soluble egg antigen from *Schistosoma mansoni* has been shown to skew CD4^+^ T-cell responses in such a direction, potentially through its capacity to bind CLRs present on the surface of DCs [21–23]. Whereas *S*. *mansoni* is quite well studied, far less is known about the effect of filarial nematodes on cells of the human immune system. Lymphatic filariasis is caused by *Wuchereria bancrofti*, *Brugia malayi* and *Brugia timori* [3]. *B*. *malayi*. is found in South East Asia and has several life-cycle stages in the human host, L3 larvae, L4 larvae, adult worms and their offspring microfilaria (mf) L1 larvae [3]. Each of the developmental stages has a unique set of immunological characteristics [3,24,25] and it is the adult worm, especially the female, which is capable of skewing T-cell responses towards a Th2 phenotype [26]. The exact mechanism by which *B*. *malayi* skews the immune system is still unknown although the presence of phosphorylcholine (PC) groups on molecules derived from filarial nematodes seems to play an important role.

The most studied PC-containing component from a filarial nematode is the excretory secretory protein 62 (ES-62) from the rodent filarial nematode *Acanthocheilonema viteae*. A 77% homologous molecule has been shown to be secreted by adult *B*. *malayi* parasites [27,28]. ES-62, alternatively known as leucylamino peptidates (LAP), possesses a range of immunomodulatory activities [29,30]. Firstly, it has been shown to interfere with signaling through the T-cell and B-cell receptors rendering cells unresponsive to subsequent antigen activation [29,31,32]. Secondly, ES-62 can also interact with TLR4 thereby inhibiting MyD88-mediated signaling and prevent subsequent TLR4 signaling [33]. Thirdly, ES-62 has been shown to promote a Th2 cell-inducing phenotype in murine macrophages and DCs [34,35]. Fourthly, ES-62 has been demonstrated to induce Th2 rather than Th1 cell responses *in vivo* based on the antibody responses that were skewed towards IgG1 [36,37]. And lastly, ES-62 has been shown to have beneficial effects on the outcome of autoimmune diseases such as asthma, arthritis and lupus in mouse model systems [38–40]. Although there is very limited data on the effect of ES-62 on human cells, mast cells and Jurkat T-cells have been studied, ES-62 clearly modulated their responses to certain stimuli [29,41]. The active component of ES-62 has been identified as PC which is covalently attached to a N-linked glycan [36,42–44]. The ES-62 homologue secreted by adult *B*. *malayi* is however not the main PC-carrying molecule, here N-acetyl glycosaminyltransferase also secreted by the adult worm has the highest PC content [28].

Here we studied the effect of BmA and ES-62 on HIV-1 *cis*- and *trans*-infection. Additionally, we examined whether exposing DCs to BmA and ES-62 resulted in modulated maturation, Th-cell induction or the susceptibility of the induced Th-cells to HIV-1 infection.

## Materials and Methods

### Parasitic products

Adult *Brugia malayi* worms were purchased from TRS labs (Athens, Georgia, USA). *B*. *malayi* adult worm extract (BmA) was prepared by homogenization of adult male and female worms on ice in PBS containing 0.5% n-octyl glucoside (PBS-nOG). The homogenates were centrifuged at 12,000 g, and the insoluble pellet was extracted once more with PBS-nOG to remove any remaining soluble antigen. The supernatants were pooled and passed through a 0.45 mm filter with the protein concentration determined using the Bradford method. The BmA total protein extract was aliquoted and stored at -70°C until use at the desired concentration. Highly purified, endotoxin-free ES-62 was prepared essentially as previously described [45].

### Viruses

The HIV-1 subtype B replication competent viruses SF162 (R5) and LAI (X4) were used. SF162 is a CCR5 using virus which is a molecular cloned isolate obtained from an HIV-1 infected patient. The CXCR4 using virus LAI also represents a molecular cloned virus isolated from an HIV-1 infected patient. Viruses were passaged on CD4 enriched T-cells and tissue culture infectious dose (TCID_50_) values were determined by limiting dilutions on these cells according to the Reed and Muench method [46].

### ELISA’s

The DC-SIGN binding ELISA was performed as described [12]. In short, BmA (25μg/ml) or ES-62 (8μg/ml) were coated on an ELISA plate followed by addition of 333ng/ml DC-SIGN-Fc (R&D systems). Next, a secondary goat-anti-human-Fc HRP labelled antibody (1:1000, Jackson Immunology) was used to detect DC-SIGN-Fc bound to the plate. In the gp140 competition ELISA, 10μg/ml anti-HIV-1 gp120 antibody D7324 (Aalto BioReagents Ltd) was coated on an ELISA plate after which trimeric HIV-1 gp140 (JR-FL SOSIP.R6-IZ-D7324) was added, as previously described [47,48]. Meanwhile 333ng/ml DC-SIGN-Fc (R&D systems) was incubated with BmA (50μg/ml), subsequently the mixture was added to the gp140 coated plate. Using a secondary HRP labelled goat-anti-human-Fc antibody (Jackson Immunology) (1:1000), DC-SIGN-Fc—gp140 binding was determined [48]. The Capsid p24 ELISA was performed as standard [10]. In short, culture supernatant was added to an ELISA plate coated with 10μg/ml sheep anti-p24-specific antibody (Aalto Bio Reagents Ltd.). Next, 4ng/ml mouse anti-HIV-1-p24 alkaline phosphatase conjugate antibody (Aalto Bio Reagents Ltd.) was added followed by Lumi-phos plus (Lumigen Inc.) (development solution) according to the manufacturer's protocol. A serial dilution of *Escherichia coli*-expressed recombinant HIV-1-p24 (Aalto Bio Reagents Ltd.) functioned as the standard curve.

Cytokine concentrations were determined using a standard ELISA protocol and the following antibody pairs IL6 (CD205_-c_ 1:200 and CD205_-d_ 1:200, U-CyTech), IL10 (JES3-9D7 1:1000 and JES3-12G8 1:1000, BD Pharmingen), IL12 (20C2 1: 500 and C8.6 1:2000, own cultures) and TNF-α (MAb1 1:500 and MAb11 1:250, eBioscience).

### Cell lines and primary cell isolation

Raji DC-SIGN cells were cultured in RPMI 1640 (Invitrogen) supplemented with 10% FCS, 100U/ml penicillin and 100U/ml streptomycin (100U/ml Pen/ Strep). Peripheral blood mononuclear cells (PBMCs) were isolated from buffy coats (purchased Sanquin) obtained from healthy volunteers using ficoll-hypaque density centrifugation. PBMCs of 3 CCR5 wild-type homozygous donors were pooled and cultured in RPMI 1640 containing 10% FCS, 100U/ml Pen/ Strep, 100U/ml recombinant IL-2 (Chiron), and 2μg/ml phytohemagglutinin (PHA, Remel) was used to activate the cells. After 5 days of culture PBMCs were enriched for CD4^+^ T-cells by depleting the CD8^+^ T-cell population using CD8 dynabeads (Life Technologies) according to manufacturer’s protocol. Monocytes were isolated from PBMC as previously described [49]. Monocytes cultured in IMDM (Gibco) containing 5% FCS, 86μg/ml gentamycin (Duchefa), 500U/ml GM-CSF (Schering-Plough) and 10U/ml IL-4 (Miltenyi Biotec) for 6 days differentiated into immature DCs (iDCs). The CD4^+^ T-cell isolation MACS kit (Miltenyi Biotec, 130-091-155) was used according to manufacturer’s protocol to isolate CD4^+^ T-cells from the PBL fraction. Subsequently, the CD4^+^CD45RA^+^CD45RO^-^ naïve T-cells were isolated from the CD4^+^ T-cells using anti-CD45RO-PE (DAKO, R084301) and anti-PE beads (Miltenyi-Biotec, 130-048-801), described in detail [49].

### HIV-1 *cis*- infection

CD4^+^ enriched T-cells (2x10^5^ cells/well) were incubated with 25, 5 or 1μg/ml BmA, 4, 2, 1μg/ml ES-62 or medium (control) 2h prior to and during HIV-1 infection. HIV-1 SF-162 (TCID_50_/ml 1000) or LAI (X4) (TCID_50_/ml 200) was added and 4, 7 and 12 days post-infection HIV-1 Capsid p24 was determined in the supernatant by ELISA. Neither antigen source was shown to be toxic for iDCs (S1 Fig).

### HIV-1 *trans*-infection assay

Raji DC-SIGN cells (2x10^4^ cells/well) were pre-incubated for 2h with 5μg/ml BmA after which SF162 (R5) (TCID_50_/ml 5000) or LAI (X4) (TCID_50_/ml 2500) was added for an additional 2h. Subsequently, the cells were washed 3 times and co-cultured with 2x10^5^ cells/well CD4^+^-enriched T-cells. 4, 7 and 12 days post-infection viral outgrowth was measured by determining the supernatant Capsid p24 levels (using the p24 ELISA). HIV-1 capture/transfer by iDCs was conducted with minor modifications as to the protocol described above, where BmA antigen was tested at 500μg/ml and where the incubation period was shortened to 0.5h. These adaptations were made due to the high expression levels of DC-SIGN on iDCs and rapid receptor turnover at the cell surface.

### Assessment of DC maturation

Between 25-40x10^3^ iDCs were cultured in a 96 well plate with 1, 2 or 4μg/ml ES-62 or 10, 25 or 75μg/ml BmA in the presence of 1.0 or 100ng/ml LPS for 24h. The supernatant was stored at -20°C and the IL-6, IL-10, IL-12 and TNF-α concentrations determined by ELISA. 40x10^4^ monocytes were cultured (24 well plate) in IMDM containing 5% FCS, 86μg/ml gentamycin (Duchefa), 500U/ml GM-CSF (Schering-Plough) and 10U/ml IL-4 (Miltenyi Biotec). After 6 days monocytes had differentiated into iDCs and half of the medium was replaced with medium containing 1, 2 or 4μg/ml ES-62 or 10, 25 or 75μg/ml BmA in the presence of 1.0 or 100ng/ml LPS and where 48h later the cells were FACS-analysed for CD86, CD83 and HLA-DR expression.

### DC-T-cell outgrowth model system

Donor A iDCs were matured with 100ng/ml LPS (Sigma Aldrich) or 100ng/ml LPS and 10μM PgE_2_ (Sigma Aldrich) in the absence or presence of 25μg/ml BmA or 2–4μg/ml ES-62. Matured DCs were washed 3 times, subsequently 5x10^3^ cells were co-cultured with 2x10^4^ CD4^+^CD45RA^+^CD45RO^-^ T-cells from donor B and 10pg/ml Staphylococcus enterotoxin B (Sigma-Aldrich) in IMDM, 10% FCS, 86μg/ml gentamycin (Duchefa). At day 5 and 7 the cells were split and the medium was supplemented with 20U/ml IL-2. At day 8, 5x10^4^ cells/well were plated and infected with SF162 (R5) (TCID_50_/ml 1000) or LAI (X4) (TCID_50_/ml 200). Cells were re-stimulated with 10ng/ml PMA (Sigma-Aldrich), 1μg/ml ionomycin (Sigma-Aldrich), 10μg/ml brefeldin A (Sigma-Aldrich) and 0.1μg/ml T1294 (Pepscan Therapeutics BV) for 6h day 5 and 7 post-infection. Subsequently, the cells were fixed in 3.7% formaldehyde and stored for no more than 1 week at 4°C in FACS buffer (PBS+ 2%FCS) for flow cytometry analysis. CD4^+^ T-cells are the major cell type being analysed here since the culture conditions used are non-permissive for iDC survival. HIV-1 infection is determined at the optimal time-point which varies between donor and virus and where cell viability is high (>50%).

### Flow cytometry

To determine CD86, CD83 and HLA-DR on DCs the cells were washed twice using FACS buffer and were subsequently stained using CD86-PE (IT2.2, BD Biosciences), CD83-APC (HB15e, Biolegend) and HLA-DR-PerCp (L243, BD Biosciences) for 30min at 4°C in FACS buffer. Cells were washed once more with FACS buffer and were analysed on a FACS Canto II apparatus. To assess the capacity of T-cells to produce cytokines/ chemokines, cells were permeabilized with PermWash (BD Pharmingen) according to manufacturer’s protocol to stain for intracellular markers. Cells were stained for p24-RD1 (Beckman Coulter, KC57-RD1) and IFN-γ-FitC (4S.B3), IL2-PerCp-Cy5.5 (MQ1-17H12), IL-4-APC (MP4-25D2), TNF-α-PE-CF594 (MAb11), Mip-1β-AlexaFluor700 (D21-1351) (all from BD Bioscience) for 30min at 4°C in PermWash. Next, these cells were washed once with PermWash, resuspended in FACS buffer (PBS+ 2%FCS) and measured on a FACS Canto II apparatus. The histograms depict all T-cells capable of producing a certain cytokine/ chemokine at the optimal time point. The optimal time point was determined by the percentage of live cells (>50%) and the level of HIV-1 infection, which varied per donor and per virus between day 5 and 7.

### Statistics

The unpaired student T tests were used except in figures demonstrating relative levels of infection. Here the paired student T test was utilized.

## Results

### BmA but not ES-62 blocks HIV-1 *trans*-infection of CD4^+^ T-lymphocytes

**Fig 1:**
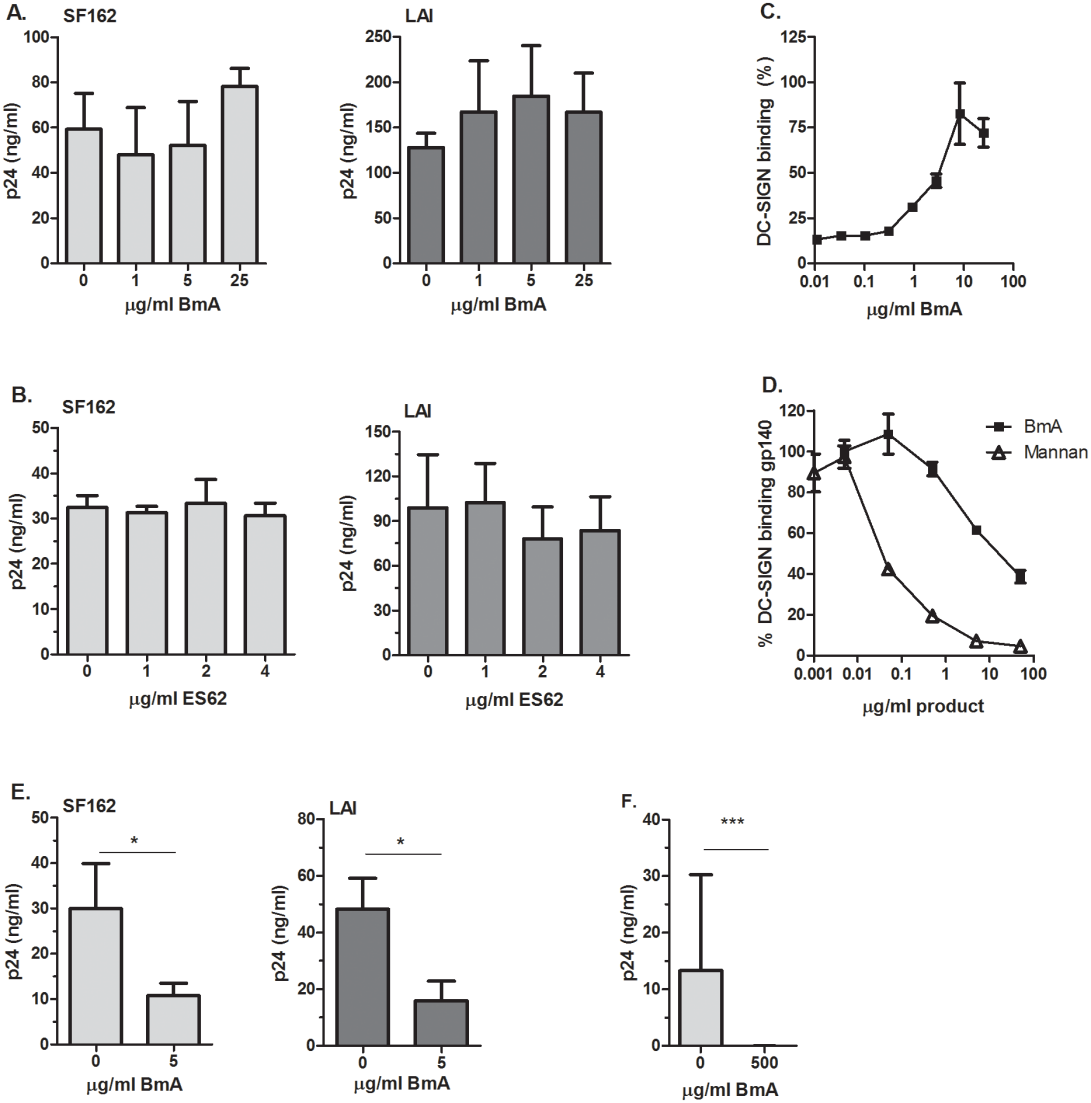
BmA inhibits HIV-1 *trans*-infection but not *cis*-infection whereas ES-62 does not affect either. The effect of 1, 5 and 25μg/ml BmA (**A**) and 1, 2 and 4μg/ml ES-62 (**B**) on HIV-1 SF162 (R5) and LAI (X4) *cis*-infection of CD4^+^ enriched T-cells determined by viral outgrowth (supernatant p24 levels). (**C**) BmA (max. 25μg/ml) was coated on to an ELISA plate after which DC-SIGN-Fc binding was determined (DC-SIGN binding ELISA). The highest OD was set to 100% binding demonstrating a dose-response curve. (**D**) The capacity of DC-SIGN-Fc, pre-incubated with BmA (max. 50μg/ml) or mannan (max. 50μg/ml), to bind a gp140 coated ELISA plate was determined (gp140 competition ELISA). The highest OD was set to 100% implying that 0.001μg/ml of BmA and mannan do not block DC-SIGN-Fc binding to gp140. (**E**) Raji-DC-SIGN cells were incubated with 5μg/ml BmA before addition of SF162 (R5) or LAI (X4). Subsequently viral outgrowth (supernatant p24 levels) was monitored in Raji DC-SIGN-CD4^+^ enriched T-cells co-cultures. (**A-E**) Representative experiment shown where all data points were performed in triplicate and with each experiment performed at least 3 times (* p<0.05). (**F**) iDCs were incubated with medium or 500μg/ml BmA before washing and the addition of SF162 (R5). Viral outgrowth (supernatant p24 levels) was monitored in iDC-CD4^+^ enriched T-cell co-cultures. Three donors were tested in duplicate and all results were combined (***p<0.001).

Previous studies have shown that an array of pathogen-derived glycoproteins bind DC-SIGN [9]. Here we assessed whether the filarial nematode derived products BmA and ES-62 had similar properties in binding this specific CLR. First we examined their potential to modulate HIV-1 *cis*-infection of CD4^+^ enriched T-cell blasts (CD8^+^ depleted T-cell blasts). These cells were incubated with 1, 5 or 25μg/ml BmA after which the CCR5-using HIV-1 virus SF162 or the CXCR4-using virus LAI was added. Viral outgrowth of SF162 (R5) and LAI (X4), determined by the presence of HIV-1 capsid protein (p24) in the supernatant, was unaffected by the presence of BmA (Fig 1A). Similarly, SF162 (R5) and LAI (X4) outgrowth in CD4^+^ enriched T-cells exposed to 1, 2 or 4μg/ml ES-62 was also unaffected (Fig 1B). This demonstrates that neither BmA nor ES-62 has the capacity to prevent HIV-1 binding to CD4, CCR5 or CXCR4.

Next we assessed the capacity of BmA and ES-62 to bind DC-SIGN, which plays a prominent role in HIV-1 *trans*-infection. Utilizing a DC-SIGN binding ELISA we found that DC-SIGN-Fc bound dose-dependently to increasing concentrations of BmA (Fig 1C) whilst DC-SIGN-Fc did not bind to ES-62 (data not shown). To gain insight into the potential of BmA to prevent HIV-1 *trans*-infection we performed a gp140 competition ELISA in which trimeric gp140, mimicking HIV-1’s natural envelope protein, is coated to a plate after which DC-SIGN-Fc pre-incubated with BmA was added. Limiting dilutions of BmA demonstrated a dose-dependent inhibition of DC-SIGN-Fc binding to the gp140 coated plate, although to a lesser extent than mannan which potently binds DC-SIGN and inhibit HIV-1 capture (Fig 1D).

To address whether BmA is capable of preventing HIV-1 *trans*-infection at the cellular level Raji DC-SIGN cells were incubated with BmA prior to addition of HIV-1 SF162 (R5) or LAI (X4). Viral outgrowth of both viruses in Raji DC-SIGN-CD4^+^-enriched T-cell blast co-cultures was significantly reduced (p = 0.033 and p = 0.012, respectively) when Raji DC-SIGN cells were pre-incubated with 5μg/ml BmA, indicating that BmA can block HIV-1 *trans*-infection (Fig 1E). Similarly, when testing iDCs from three different donors with a high concentration of BmA (500μg/ml) we found 100% inhibition in all three donors of HIV-1 capture and transfer (Fig 1F). In conclusion, neither BmA nor ES-62 can block *cis*-infection of CD4 enriched T-cells while BmA can bind DC-SIGN and block HIV-1 capture and transfer.

### BmA and ES-62 do not alter DC maturation

**Fig 2:**
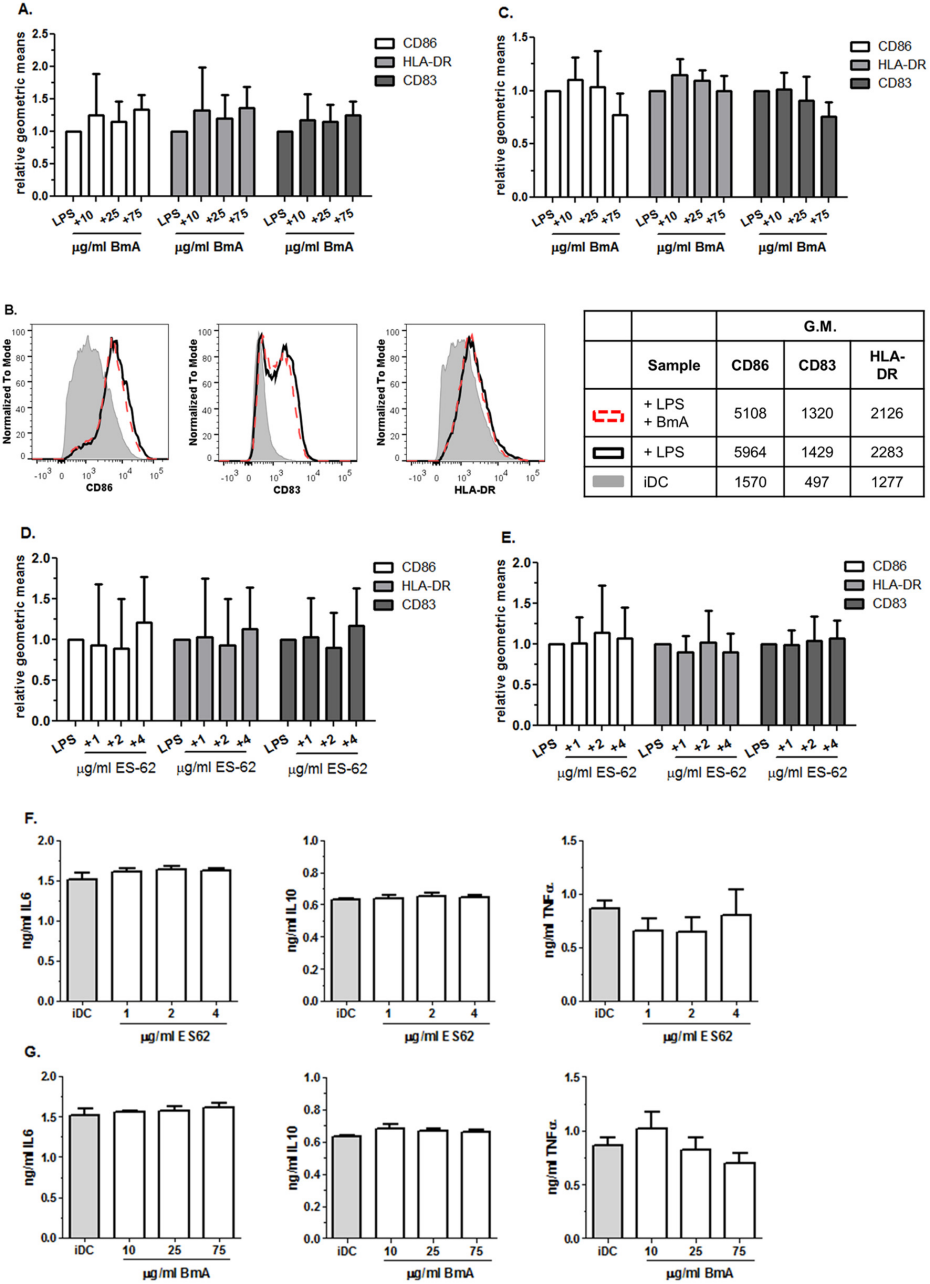
BmA and ES-62 do not alter surface expression of co-stimulatory molecules or cytokine production. (**A**) The geometric mean of CD86 (white), HLA-DR (grey) and CD83 (dark grey) on DCs exposed to 100ng/ml LPS and 10, 25 or 75μg/ml BmA expressed relative to the geometric mean found on cells exposed to 100ng/ml LPS only. (**B**) A representative FACS plot demonstrating the expression of CD86 (left), CD83 (middle) and HLA-DR (right) on iDC (grey), DC exposed to 100ng/ml LPS (black) and DC exposed to 100ng/ml LPS + 25μg/ml BmA (dashed). (**C**) The geometric mean of CD86 (white), HLA-DR (grey) and CD83 (dark grey) on DCs exposed to 1ng/ml LPS and 10, 25 or 75μg/ml BmA expressed relative to the geometric mean found on cells exposed to 1ng/ml LPS only. (**D**) The geometric mean of CD86 (white), HLA-DR (grey) and CD83 (dark grey) on DCs exposed to 100ng/ml LPS and 1, 2 or 4μg/ml ES-62 expressed relative to the geometric mean found on cells exposed to 100ng/ml LPS only. (**E**) The geometric mean of CD86 (white), HLA-DR (grey) and CD83 (dark grey) on DCs exposed to 1ng/ml LPS and 1, 2 or 4μg/ml ES-62 expressed relative to the geometric mean found on cells exposed to 1ng/ml LPS only. IL6 (left), IL10 (middle) and TNFα (right) cytokine production by DCs exposed to 100ng/ml LPS and 4μg/ml ES-62 (**F**) or 100ng/ml LPS and 75μg/ml BmA (**G**). (**A, C-E**) Data of three independent experiments combined (mean and SD), (**F** and **G**) representative of 2 independent experiments performed in triplicate.

In previous studies both BmA [50] and ES-62 [34,35] have been shown to modulate murine macrophage and/or DC responses. Here we examined whether BmA or ES-62 were capable of inducing, facilitating or altering human DC maturation in the context of co-stimulatory molecules as well as cytokine production. To this end, DCs were matured under optimal conditions (100ng/ml LPS) and suboptimal conditions (1ng/ml LPS) in the presence of 10, 25 or 75μg/ml BmA. We demonstrate that the addition of BmA does not alter the cells capacity to upregulate co-stimulatory molecules CD86, CD83 and HLA-DR, whether the cells are matured under suboptimal or optimal conditions by expressing the geometric mean found in conditions with BmA as a relative in- or de-crease compared to the LPS control condition (Fig 2A–2C). Similarly, when maturing iDCs in the presence of 100ng/ml LPS and 1, 2 or 4μg/ml ES-62 we also did not observe differences in the level of CD86, CD83 or HLA-DR compared to the LPS control condition (Fig 2D and 2E). Furthermore, BmA and ES-62 could not upregulate CD86, CD83 or HLA-DR on iDCs (S2 Fig).

To determine effects on cytokine production, iDC were exposed to 10, 25 or 75μg/ml BmA in absence or presence of 100ng/ml LPS. The supernatant was analyzed for IL-6, IL-10, IL-12 and TNF-α. IL-6, IL-10 and TNF-α were only produced when cells were stimulated with 100ng/ml LPS and the amount produced, 1.5ng/ml, 0.6ng/ml and 0.9ng/ml respectively was not affected by the presence of 10, 25 or 75μg/ml BmA (Fig 2G). Similarly, 1, 2 or 4μg/ml ES-62 did not affect the amount of IL-6, IL-10 and TNF-α that was produced (Fig 2F). IL-12 was not produced to detectable amounts in any condition (data not shown). Collectively our data suggest that neither BmA nor ES-62 can alter LPS induced up-regulation of co-stimulatory molecules or alter cytokine production.

### BmA-exposed DCs do not induce Th-cells with an altered susceptibility to HIV-1

**Fig 3:**
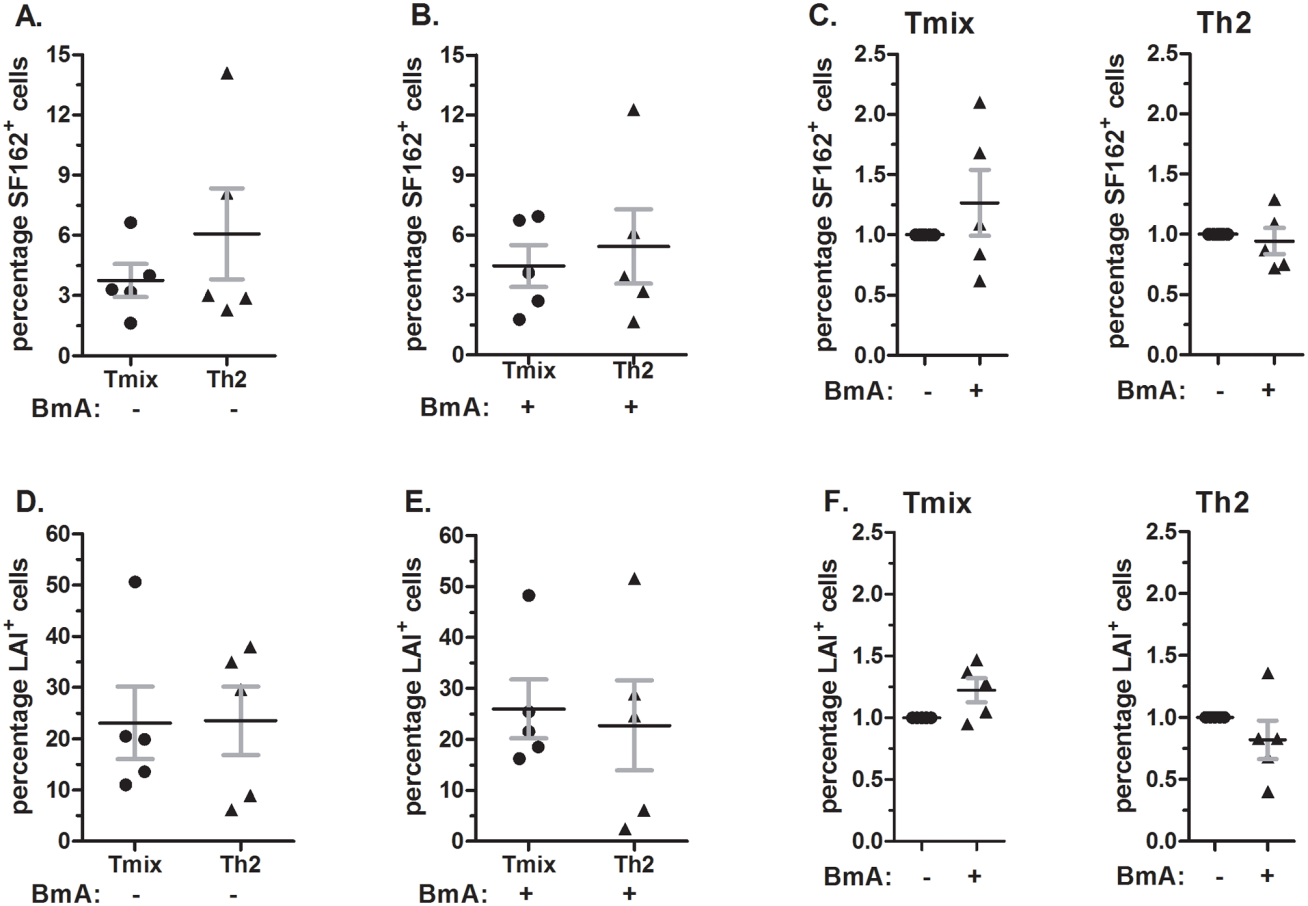
Exposing DCs to BmA does not alter the HIV-1 susceptibility of the induced Th-cell population. (**A, D**) Percentage SF162^+^ (**A**) or LAI^+^ (**D**) cells in T_mix_ and Th2 cell cultures induced by unexposed DCs. (**B, E**) Percentage SF162^+^ (**B**) or LAI^+^ (**E**) cells in T_mix_ and Th2 cell cultures induced by BmA exposed DCs. (**C, F**) The relative expression of SF162^+^ (**C**) and LAI^+^ (**F**) in T_mix_ and Th2 cell cultures induced by BmA exposed DCs compared to the expression in Th-cell cultures induced unexposed DC. (**A-F**) Depicted are mean values and SEM of 5 independent experiments using 5 different DC-T cell donor combinations.

Although BmA and ES-62 do not alter DC maturation described above there are other factors that can influence the T-cell outgrowth in DC-T-cell co-cultures which potentially alters T-cell HIV-1 susceptibility. For instance, the level of T-cell activation has been associated with the number of HIV-1 restriction factors that are up regulated [20]. In order to determine whether the HIV-1 susceptibility of Th-cells induced by DCs exposed to BmA or ES-62 is altered, we established an *in vitro* cell system. Here iDCs were matured in Th1/Th2 mixed (LPS) or Th2 (LPS+ PgE_2_) promoting conditions in the absence or presence of BmA or ES-62. Co-culturing mature Staphylococcus Enterotoxin B-loaded DCs from donor A with naïve CD4^+^CD45RA^+^ T-cells from donor B for 8 days resulted in the respective Th-cell cultures, which were subsequently infected with HIV-1 SF162 (R5) or LAI (X4). Here we focussed on the effect of BmA on Th1/Th2 mixed (T_mix_) and Th2 cell cultures as these are expected to be preferentially modulated by parasite extracts. In the T_mix_ and Th2 cell cultures induced by unexposed DCs we found on average 3.8% ±0.82 and 6.1% ±2.3 SF162 (R5) infected T-cells respectively (p = 0.36) (Fig 3A). Similar percentages of SF162 (R5) infected cells were observed in T_mix_ and Th2 cell cultures induced by BmA exposed DCs, 4.4% ±1.0 and 5.4% ±1.9 respectively (p = 0.66) (Fig 3B). To determine whether BmA affected SF162 (R5) susceptibility of T-cell cultures, we expressed the percentage of SF162^+^ T-cells induced by BmA exposed DCs relative to the percentage of SF162^+^ T-cells in cultures induced by unexposed DCs (set at 1.0). This revealed that both in T_mix_ and Th2 cell cultures the level of SF162 (R5) infection is similar, p = 0.39 and p = 0.63, respectively (Fig 3C).

In LAI (X4) infected T_mix_ and Th2 cell cultures we find on average similar percentages of LAI^+^ cells 23.1% ±7.1 and 23.5% ±6.7, respectively (p = 0.97), when these cultures were induced by unexposed DCs and 26.0% ±5.8 and 22.7 ±8.8 LAI^+^ cells, respectively (p = 0.77), when induced by BmA-exposed DCs (Fig 3D and 3E). Again, when we express the level of infection found in T-cell cultures induced by BmA-exposed DCs relative to the level of infection in T-cell cultures induced by unexposed DCs we found that the level of infection is similar in T_mix_ and Th2 cell population, p = 0.08 and p = 0.31, respectively (Fig 3F). The difference in the level of infection obtained by SF162 (R5) and LAI (X4) can be explained by the difference in efficiency by which R5 and X4 HIV-1 viruses infect CD4^+^ T-cells *in vitro* [51]. Collectively, our data demonstrate that exposing DCs to BmA does not result in T-cell cultures with an altered susceptibility to HIV-1 infection.

### Th-cell cultures induced by BmA- or ES-62 exposed DCs do not have a more pronounced Th2 phenotype

As *B*. *malayi* infection has been previously shown to skew T-cell responses towards a Th2 cell profile in a murine model, we evaluated the cytokine/chemokine (IFN-γ, IL-4, IL-2, TNF-α, MIP-1β) profile of the Th-cell cultures induced by BmA exposed DCs [52]. All cells produced IL-2 and TNF-α under all circumstances (data not shown).

**Fig 4:**
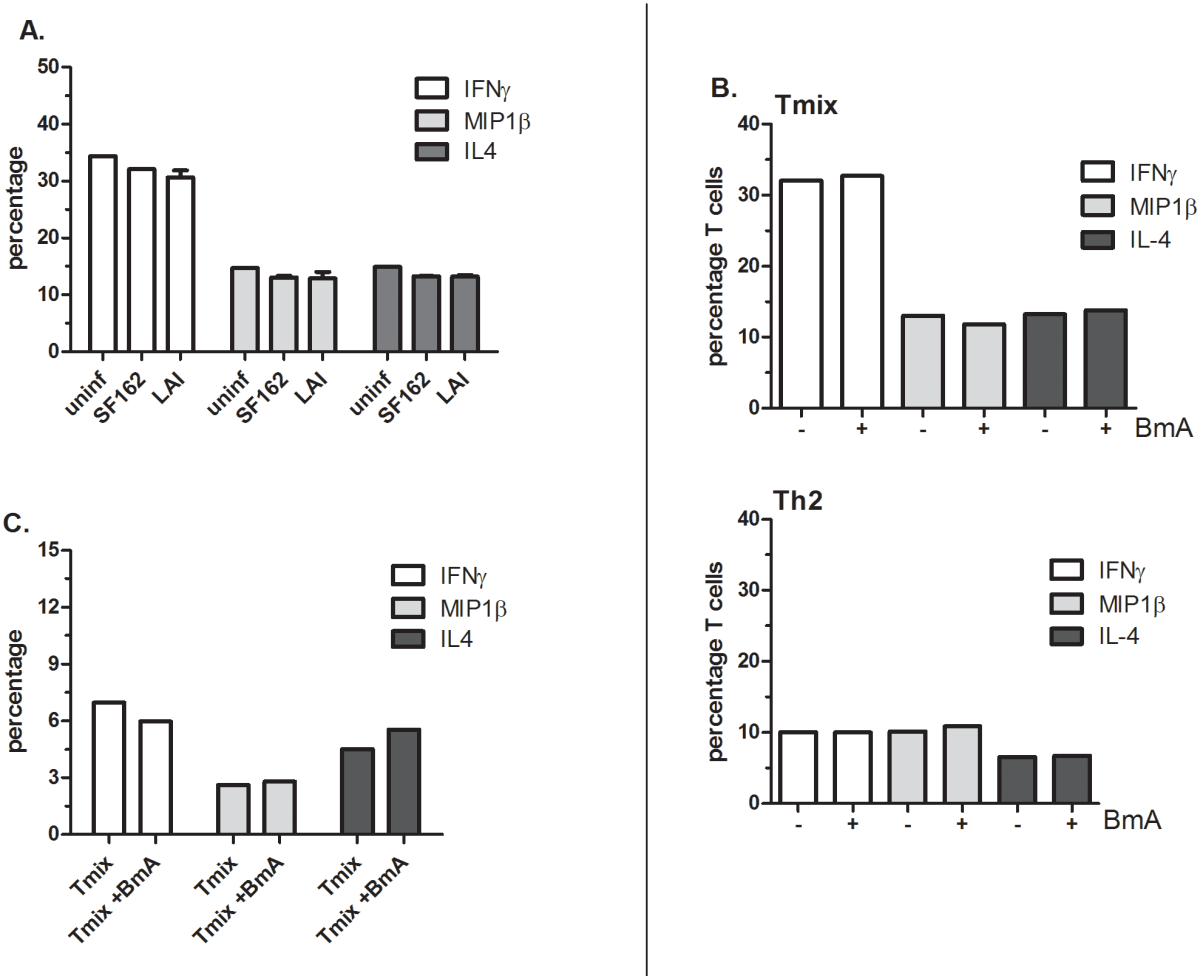
Exposure of DCs to BmA does not alter Th-cell cytokine/chemokine expression profiles. (**A**) Percentage of IFNγ, MIP-1β and IL-4 producing T-cells in uninfected (1^st^ bar), SF162 (R5) (2^nd^ bar) or LAI (X4) (3^rd^ bar) infected Th-cell cultures. The figure is representative for both T_mix_ and Th2 cell cultures. (**B**) Percentage of IFNγ, MIP-1β and IL-4 producing T-cells in a T_mix_ (upper figure) and a Th2 (lower figure) cell culture induced by DCs unexposed (1^st^, 3^rd^, 5^th^ bar) or exposed to 25μg/ml BmA (2^nd^, 4^th^, 6^th^ bar). (**C**) IFNγ, MIP-1β and IL-4 producing T-cells in a T_mix_ cell culture induced by DCs unexposed or exposed to 75μg/ml BmA. (**A-C**) Representative figure of an experiment performed at least 3 times in (**A**) the conditions where virus was added were performed in duplicate.

First we demonstrated that HIV-1 SF162 (R5) or LAI (X4) infection did not influence the capacity of the Th-cells to produce IFN-γ, MIP-1β or IL-4 (Fig 4A). Subsequently, we determined the effect of exposing DCs to 25μg/ml BmA on the capacity of the induced Th-cell cultures to produce IFN-γ, IL-4 and MIP-1β. Both in T_mix_ and Th2 cultures we did not find differences in the level of IFN-γ, IL-4 and MIP-1β when BmA was added to maturing DCs (Fig 4B). Even when the BmA concentration that DCs were exposed to was increased to 75μg/ml no effect on the percentage of cells producing IFN-γ, IL-4 and MIP-1β in the induced T_mix_ cell cultures was observed (Fig 4C).

**Fig 5:**
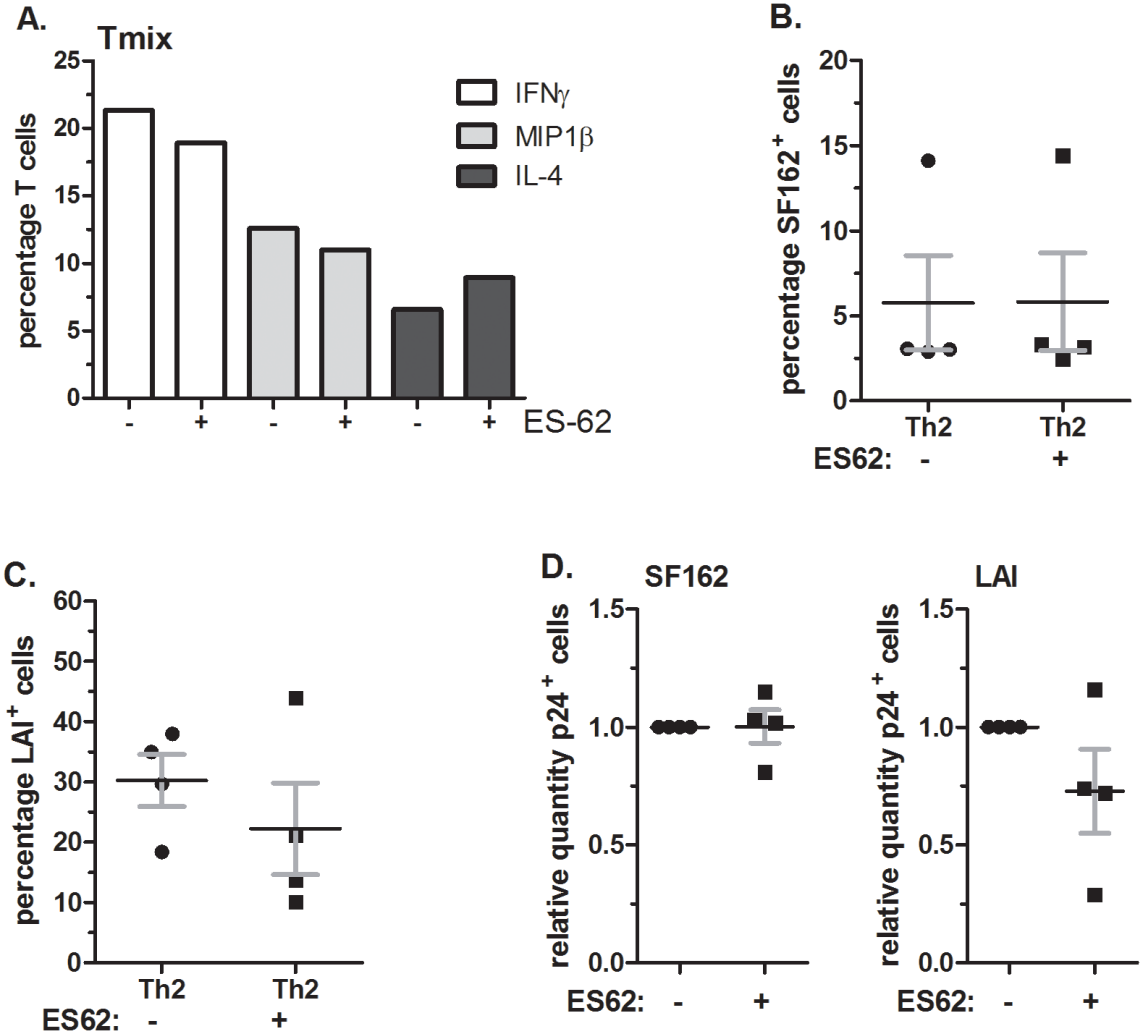
Exposing DCs to ES-62 does not alter the HIV-1 susceptibility of the induced Th-cell population nor their cytokine/chemokine profiles. (**A**) Percentage of IFN-γ, IL-4 and MIP-1β producing T cells in Th2 cell cultures induced by DCs unexposed or exposed to 2–4μg/ml ES-62. (**B**) Percentage SF162^+^ T-cells found in Th2 cell cultures induced by unexposed DCs (left) or DCs exposed to 2–4μg/ml ES-62 (right). (**C**) Percentage LAI^+^ T-cells found in Th2 cell cultures induced by unexposed DCs (left) or DCs exposed to 2–4μg/ml ES-62 (right). (**D**) The relative level of SF162 (R5) (left) and LAI (X4) infection (right) in Th2 cultures induced by DCs exposed to 2–4μg/ml ES-62 compared to the level found in Th2 cell cultures induced by unexposed DCs. (**A**) A representative figure of an experiment performed at least 3 times; (**B-D**) mean values and SEM of 4 independent experiments using 4 different DC-T cell donor combinations.

Similarly, DCs exposed to ES-62 did not induce T-cell cultures that have an altered cytokine/chemokine profile (Fig 5A). Moreover, as with BmA, ES-62 exposed DCs do not alter the T-cells capacity to support HIV-1 infection. The average percentage of SF162^+^ T-cells were very similar in cultures induced by unexposed or ES-62 exposed DCs, 5.7% ± 2.8 and 5.8% ±2.9 respectively (Fig 5B) and no effect of ES-62 on the percentage of infected T-cells was observed (p = 0.97, Fig 5D left). The average percentage of LAI^+^ T-cells in Th2 cultures induced by unexposed DCs was slightly higher, 30% ±4.3 than the percentage found in Th2 cultures induced by ES-62-exposed DCs, 22.2% ±7.6 (p = 0.39) (Fig 5C). However, even when determining the relative difference between the percentage of LAI^+^ cells in cultures induced by unexposed versus ES-62 exposed DCs no significance was found (p = 0.22) (Fig 5D right). In summary, both BmA and ES-62 exposed DCs do not induce Th2 skewing in our model system, potentially explaining why HIV-1 infection is unaffected in the T-cell cultures induced by DCs exposed to the parasite extract.

## Discussion

Parasitic helminths have co-evolved with the human host for millions of years during which they have acquired immunomodulatory properties. Here we aimed to determine whether BmA from adult *B*. *malayi* and ES-62 from *A*. *viteae* had immunomodulatory properties that influence HIV-1 infection. We demonstrate that BmA, but not ES-62, can potently bind DC-SIGN and inhibit HIV-1 *trans*-infection of CD4^+^ enriched T-cells, whilst neither antigen can block *cis*-infection. We identified using an *in vitro* DC-T-cell outgrowth assay that neither BmA nor ES-62 had the capacity to modulate DC maturation (CD86, CD83, HLA-DR, IL-6, IL-10, IL-12 and TNF-α) or the phenotype of the induced of Th-cells (IFN-γ, IL-4 and MIP-1β). In addition, the HIV-1 susceptibility of the Th-cells induced by DCs exposed to either BmA or ES-62 remained unaltered.

The major studies performed to date regarding altered responses of antigen presenting cells and subsequent Th2 skewing by BmA have been performed in mouse model systems. One study demonstrates that adherent peritoneal exudate cells (PEC), isolated from mice that had 6 adult female *B*. *malayi* worms *intra*-peritoneally implanted, have the capacity to induce Th2 cell responses when co-cultured with naïve T-cells [52]. Approximately 80% of PEC is composed of macrophages suggesting that they are the cell type responsible for Th2 skewing in this system whereas we used DCs [52,53]. Another study demonstrates the capacity of BmA to induce TNF-α, IL-1β and nitric oxide (NO) production by murine macrophages in a TLR4 dependent manner [50]. These effects were attributed to the presence of a LPS-like molecule derived from the intracellular bacterium *Wolbachia* that plays an essential role in the survival and fertility of filarial nematodes. Human filarial nematodes are infected with *Wolbachia* whilst *A*. *viteae* is not. Indeed *A*. *viteae* lysates could not induce cytokine or NO production by murine macrophages [3,50,54]. The potency of *Wolbachia* in altering human immune responses was emphasized by a recent study which demonstrated that the major surface component of *Wolbachia* in filarial nematodes can signal through TLR2 and TLR4 thereby inducing cytokine production in human embryonic kidney cells, primary murine macrophages and DCs as well as human PBMCs [55]. When we examined our BmA batch for the presence of LPS or an LPS-like molecule using a Limulus amebocyte lysate (LAL) assay we found it did not contain such a molecule (data not shown), thereby contributing to an explanation for the lack of changes to iDC, maturing DCs and the subsequently induced Th-cell populations. It should be noted that mice do not express DC-SIGN but eight homologues of which the SIGNR family resembles DC-SIGN most closely. However, thus far no homologue with similar glycan recognition, signaling and cell distribution as DC-SIGN has been identified in mice, providing another potential explanation for the discrepancy between our human data and reported mouse data [56]. It has also been demonstrated that PBMCs from patients with pathological lymphedema show enhanced transendothelial migration when stimulated with BmA compared to PBMCs from patients with an asymptomatic filarial nematode infection indicating another potential BmA mediated mechanism of immune-pathology [57].

A less complex explanation for the inability of BmA to alter DC maturation is that the components involved in these processes are present in different stages of the *B*. *malayi* life cycle. Although it has been shown that the female adult worm in particular was involved in inducing Th2 cell responses [26], a study by Semnani et al. revealed that the microfilaria (mf) L1 larvae can induce IL-6 and TNF-α production while impairing IL-10 and IL-12 production by human DCs [58]. Additionally, recent studies on the secretome of *B*. *malayi* compared the excretory secretory (ES) fractions with homogenized adult worm (BmA) and found that the ES fractions contained much higher levels of the PC-containing components which are known to have many immunomodulatory properties [28]. Therefore, the concentration of the immunomodulatory components in BmA may be too low rather than that the adult worm is not capable of altering DC maturation and subsequent Th-cell induction. Nevertheless, ES-62 is a purified glycoprotein containing PC groups and this molecule neither altered DC maturation nor Th-cell induction in our DC-T-cell outgrowth model.

ES-62 derived from the rodent filarial nematode *A*. *viteae* has been well studied. In mouse model systems it has been shown to alter DC maturation, induce Th2 cell responses and reduce the severity of numerous auto-immune diseases [29,31–39]. However, one study on human primary mast cells demonstrated that FcεRI receptor responses were inhibited by ES-62 following its complexing with TLR4, which resulted in protein kinase C-α sequestration and degradation [41]. Additionally, ES-62 can inhibit pro-inflammatory cytokine production in synovial membranes and fluid from patients with rheumatoid arthritis (*ex vivo*) which is driven by macrophages [45]. Our data indicate that ES-62 has no effect on modulating human DC responses nor the Th-cell phenotype they induce which suggests that the product can be used for other immunomodulatory activities without affecting T-cell skewing or modulating viral replication in HIV-1 infected individuals. Albeit, we cannot exclude the possibility that our experimental set up may not have been optimal to detect the effect of ES-62. Previous studies have demonstrated that the effect of ES-62 is biphasic with initially a small activating effect observed followed by inertia for subsequent stimuli [59]. In our system, ES-62 was added simultaneous with the LPS stimulus, which potentially prevented ES-62 from inducing inertia despite the long incubation period (48 hours).

The active components of the glycoprotein ES-62 are the attached PC groups. Some studies in mice have demonstrated that proteins with coupled PC mimicked the results obtained by addition of ES-62 [42,43,60]. PC is not unique to parasitic helminths and is present on many different pathogens where it appears to play an important role in their survival [61]. As for the interaction of PC with the human immune system there is very limited evidence that the extensive effects found in rodents can be directly translated to humans. There are some examples described in the literature where molecules containing PC groups, which have immunomodulatory properties similar to those seen with ES-62. However, it is still unclear as to whether these effects can be attributed to the presence of the PC group. For example, the lyso-sphingolipid sphingosylphosphorylcholine (SPC) found in high density lipoproteins is capable of up-regulating CD86, CD83 and HLA-DR on human DCs as well as inducing the production of IL-12 and IL-18 [62]. Secondly, oxidized 1-palmitoyl-2-arachidoyl-*sn*-glycero-3-phosphorylcholine (OxPAPC) has been shown to bind TLR4 thereby inhibiting DC maturation with LPS, as measured by up-regulation of co-stimulatory molecules CD86, CD83, HLA-DR and several others [63]. Remarkably only the oxidized form of PAPC possessed this capacity indicating that potentially not all effects are due to the PC group [63]. Lastly, miltefosin, a phosphorylcholine esther of hexadecanol is a medicine used for the treatment of visceral leishmaniasis in HIV-1 co-infected individuals which has been shown to reduce HIV-1 replication in CD4^+^ T-cells that was, in part, caused by the onset of IFN type I production by DCs [64]. These divergent results suggest that in humans probably the context in which the PC group is present is important for the subsequent elicited immune responses.

From epidemiological data and studies using human material it is still relatively unknown what effects filarial nematodes will have on HIV-1 infection *in vivo*. One study demonstrates that the prevalence of *W*. *bancrofti* in HIV-1-infected and uninfected individuals is similar [65] while another study by Gallagher et al. found that HIV-1^+^ women were more likely to be co-infected with a filarial nematode. Since HIV-1 infection via sexual transmission is acquired later in life than lymphatic filariasis Gallagher et al. speculated that filarial nematode infections result in an increased susceptibility to HIV-1 infection [66]. Additionally, a study by Nielsen et al. demonstrated that the HIV-1 viral load, CD4^+^ cell percentage and CD4^+^/CD8^+^ cell ratio was similar in HIV-1^+^ and HIV-1^+^ *W*. *bancrofti*^+^ individuals [67] while a study by Gopinath et al. found that CD8^+^-depleted PBMCs from patients treated for lymphatic filariasis had a reduced susceptibility to HIV-1 R5 viruses compared to CD8^+^-depleted PBMCs from the same patients when they were untreated [68]. However, they also observed that the level of HIV-1 infection in CD8^+^-depleted PBMCs from healthy controls was similar to the level of infection in cells from filarial nematode infected patients suggesting that the reduced level of infection is induced by the treatment regimen rather than the death of the filarial nematodes [68]. Good *in vitro* model systems using human cells are therefore required to identify what role parasites and their antigens have on modulating HIV-1 infection.

We have shown here that BmA can potently bind DC-SIGN and block HIV-1 *trans*-infection of CD4^+^ enriched T-cells and thereby potentially play a role in modulating HIV-1 infection or replication in exposed or infected individuals, respectively. This could be pertinent given the presence of filarial nematodes within the afferent lymph vessels close to lymph nodes. Once the DC-SIGN binding product has been identified it may be possible to speculate on the physiological relevance of the concentrations we have used. Additionally, the patient’s worm load, which is highly variable, will likely be a major determinant in the concentration the DC-SIGN binding component will reach *in vivo*. From the results obtained in our *in vitro* DC-T-cell model system it seems that BmA and ES-62 do not induce beneficial, or more importantly, undesirable effects on HIV-1 infection or replication of/in Th-cells induced by DCs exposed to these parasite components. Determining the effects of other pathogen antigen sources or antigens from other stages of *B*. *malayi’s* life-cycle on modulating immune responses and affecting HIV-1 infection will provide a more complete picture in indicating what consequences parasite co-infections have on modulating HIV-1 infection.

## Supporting Information

S1 Fig Both BmA and ES-62 are non-toxic for iDCs. Immature dendritic cells were exposed to 100ng/ml LPS in combination with various concentrations of BmA (A) or ES62 (B) for 48h. Subsequently the cells were analyzed by FACS. Depicted is the percentage of cells in the live-gate.
(TIF)

S2 Fig Neither BmA nor ES-62 induces co-stimulatory molecules on iDCs. (**A**) Depicted is the expression of CD86 (left), CD83 (middle) and HLA-DR (right) on iDC (grey filled) and iDC exposed to 10μg/ml (blue, dotted), 25μg/ml (red, dashed) or 75μg/ml BmA (black, solid). (**B**) Depicted is the expression of CD86 (left), CD83 (middle) and HLA-DR (right) on iDC (grey filled) and iDC exposed to 1μg/ml (blue, dotted), 2μg/ml (red, dashed) or 4μg/ml BmA (black, solid).
(TIF)
