# Supplementary figures and images for: Brugia malayi Antigen (BmA) Inhibits HIV-1 Trans-Infection but Neither BmA nor ES-62 Alter HIV-1 Infectivity of DC Induced CD4+ Th-Cells
Source: PLoS One. 2016 Jan 25;11(1):e0146527. doi: 10.1371/journal.pone.0146527 (PMC4726616; doi:10.1371/journal.pone.0146527)

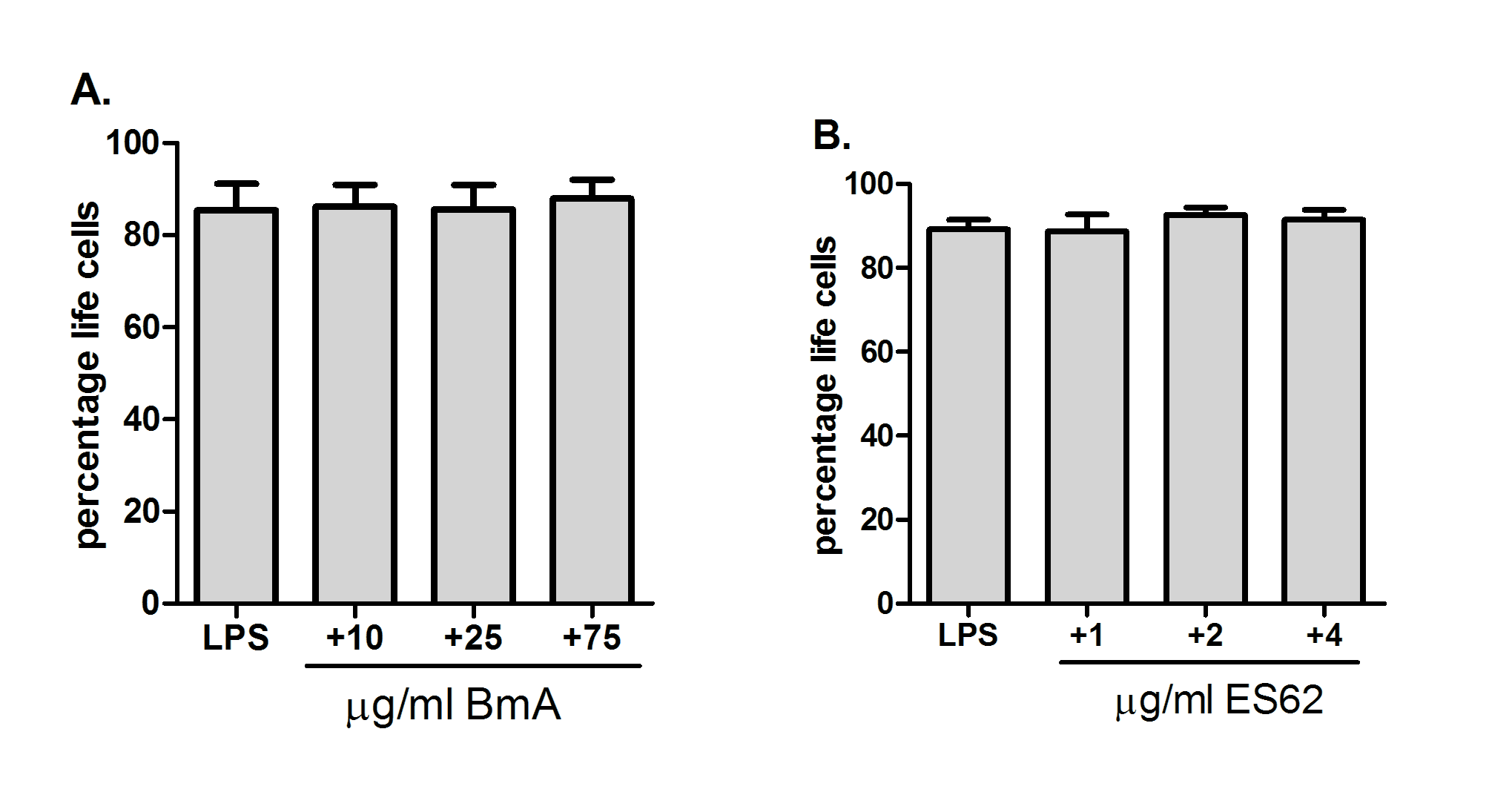

Supplement: S1 Fig — Immature dendritic cells were exposed to 100ng/ml LPS in combination with various concentrations of BmA (A) or ES62 (B) for 48h. Subsequently the cells were analyzed by FACS. Depicted is the percentage of cells in the live-gate. (TIF) [file pone.0146527.s001.tif]

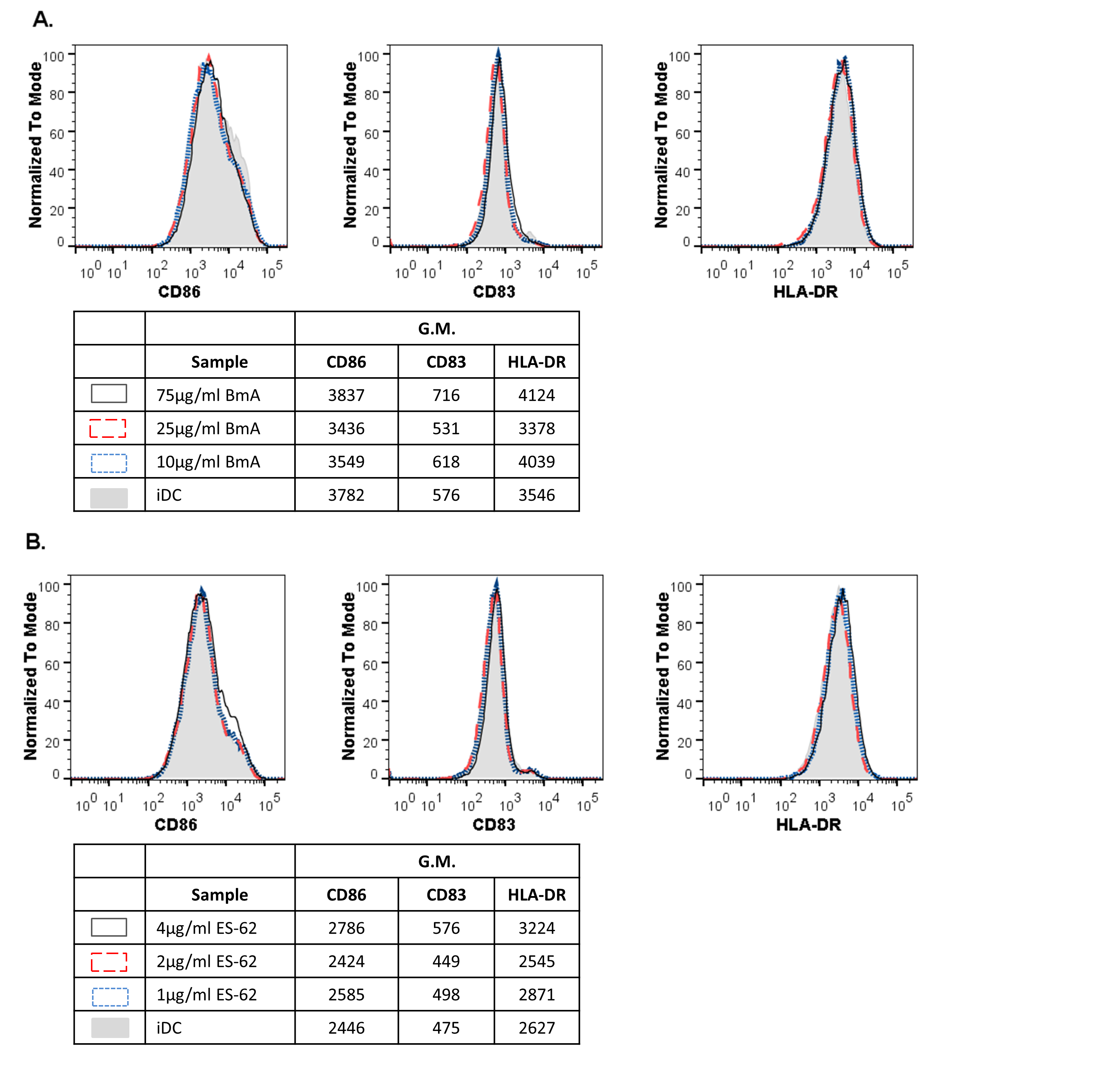

Supplement: S2 Fig — (A) Depicted is the expression of CD86 (left), CD83 (middle) and HLA-DR (right) on iDC (grey filled) and iDC exposed to 10μg/ml (blue, dotted), 25μg/ml (red, dashed) or 75μg/ml BmA (black, solid). (B) Depicted is the expression of CD86 (left), CD83 (middle) and HLA-DR (right) on iDC (grey filled) and iDC exposed to 1μg/ml (blue, dotted), 2μg/ml (red, dashed) or 4μg/ml BmA (black, solid). (TIF) [file pone.0146527.s002.tif]
